# Nutritional status of children under 5 years old in Namibia: adjusting for poor quality child anthropometry

**DOI:** 10.1017/jns.2022.67

**Published:** 2022-08-15

**Authors:** Maya S. Fujimura, Joel Conkle, Marjorie Van Wyk, Masamine Jimba

**Affiliations:** 1 Department of Community and Global Health, The University of Tokyo, 7-3-1 Hongo, Bunkyo, Tokyo, Japan; 2 David Geffen School of Medicine, Division of General Internal Medicine and Health Services Research, The University of California Los Angeles, 1100 Glendon Ave, Los Angeles, CA, USA; 3 UNICEF Namibia, Child Survival and Development, 38-44 Stein Street, Windhoek, Namibia; 4 Ministry of Health and Social Services, Government of Namibia, Private Bag 13198, Windhoek, Namibia

**Keywords:** Child anthropometry, Data quality, Namibia, PROBIT method, WHO flags

## Abstract

The poor assessment of child malnutrition impacts both national-level trends and prioritisation of regions and vulnerable groups based on malnutrition burden. Namibia has reported a high prevalence of malnutrition among children younger than 5 years of age. The present study's aim was to identify the optimal methods for estimating child stunting and wasting prevalence in Namibia using two datasets with suspected poor data quality: Namibia Demographic and Health Surveys (NDHS) (1992–2013) and Namibia Household Income and Expenditure Survey (NHIES), 2015/16. This comparative secondary data analysis used two prevalence estimation methods: WHO flags and PROBIT. WHO flags is the standard analysis method for most national household surveys, while the PROBIT method is recommended for poor quality anthropometry. In NHIES (*n* 4960), the prevalence of stunting (*n* 4780) was 30·3 and 20·9 % for the WHO flags and PROBIT estimates, respectively, and the national wasting prevalence (*n* 4637) was 11·2 and 4·2 %, respectively. The trends in nutritional status from NDHS and NHIES showed improvement across WHO flags and PROBIT until 2013; however, from 2013 to 2016, PROBIT showed smaller increases in stunting and wasting prevalence (2·5 and 0·6 percentage points) than WHO flags (6·6 and 5·0 percentage points). PROBIT identified the Khoisan ethnic group and Northern geographical regions with the highest stunting and wasting prevalence, while WHO flags identified similar prevalence across most groups and regions. The present study supports the recommendation to use PROBIT when poor data quality is suspected for constructing trends, and for targeting regions and vulnerable groups.

## Introduction

Reliable data is one of the fundamental building blocks of the overall success of a country's health system. High-quality data can be used to effectively implement national interventions and programmes for women, children, and vulnerable populations. However, poor data quality negatively impacts healthcare at individual and population levels. Over time, inconsistent data quality contributes to misleading trends that result in inaccurate interventions and health policies, concurrently obscuring the magnitude of malnutrition in a country^(1)^.

Child anthropometry is prone to measurement errors^(2)^. Many factors affect the accuracy and reliability of the data, such as human and technical errors, and lack of accessibility to anthropometric equipment. National surveys such as the DHS and Multiple Indicator Cluster Surveys flag and remove biologically implausible measurements but do not fully account for poor data quality in their survey reports^(3)^. According to the WHO–UNICEF Technical Expert Advisory Group, the data quality of child anthropometry can be assessed by seven indicators: (1) completeness, (2) sex ratio, (3) age heaping, (4) digit preference of measurements, (5) implausible *z*-score values, (6) the standard deviation (sd) of *z*-scores and (7) normality of *z*-score^(4)^. Out of these indicators, the ones which are used commonly are the biological implausibility and sd of *z*-scores. Biological implausible measurements must fall below 1 % to be considered a good quality survey. The sd of the observed mean *z*-score is an additional way to easily assess the quality, since it is subjected to the summary statistics^(5)^. For instance, the mean *z*-score sd must fall between 0·8 and 1·2 for the survey to be considered a good quality survey^(6)^.

Using an index of anthropometric data quality for the DHS, a previous study reported that the greatest variability among surveys was observed in Sub-Saharan Africa^(7)^. A number of countries have misreported the age^(8)^; height measurements tend to be inaccurate due to difficulties keeping children still, especially those under the age of 2 years^(9,10)^. DHS surveys have also shown improvement in misreporting with socio-economic development in Sub-Saharan Africa, but improvement did not occur in all countries – including Namibia^(8)^.

Namibia is an upper-middle-income country in Southern Africa. According to the Gini index, Namibia experiences one of the worst inequality levels in the world^(11)^. In 2013, Namibia DHS (NDHS) reported high malnutrition rates of stunting (24 %) and wasting (6 %) among children under age 5 years; but 12 % of child measurements were biologically implausible^(12)^. This high percentage indicates poor data quality and suggests that prevalence estimates could be inaccurate.

Over ten local languages are spoken across this ethnically and racially diverse country. Among Namibians, ethnic identity is closely tied with the language spoken. The indigenous groups are associated with certain geographical regions in the country, and many groups maintain traditional lifestyles. Many people in the Northern area of the country rely on subsistence agriculture, which has been identified with higher levels of poverty^(13,14)^.

Surveys that collect anthropometry face challenges with consistent, high-quality measurements. An existing analysis method, the PROBIT method, takes into account poor quality anthropometry and allows the use of such data, even when poor quality, is helpful for targeting interventions in resource-limited areas. Correcting for poor quality anthropometry can also help governments and international organisations to properly plan and implement nutritional aid to high-risk areas. However, although poor quality anthropometry is common, the PROBIT method is not often used to adjust for poor quality.

The objectives of the present study were twofold: (1) conducting a comparative analysis of two malnutrition prevalence estimation methods (the standard method of WHO flags and the PROBIT method) in Namibia by using the Namibia Demographic and Health Surveys and the Namibia Household Income and Expenditure Survey 2015/16; (2) analysing trends and identifying high-risk target populations and regions using these two distinct methods.

## Methods

### Measures

The nutritional status of children was defined by comparing their height, weight and age to the 2006 WHO Child Growth Standards. The malnutrition indices used in the present study were height-for-age *z*-score (HAZ) and weight-for-height *z*-score (WHZ). Children with HAZ below −2 standard deviations (sd) from the median were considered stunted. Stunting is a long-term effect of chronic malnutrition. Children with WHZ below −2 sd from the median were considered wasted. Severe acute malnutrition (SAM) was defined as WHZ below −3 sd from the median. Wasting reflects acute malnutrition.

The NHIES indicates the main language spoken in the household. The main languages were collapsed into the following categories: Khoisan, Zambezi, Otjiherero, Rukavango, Nama/Damara, Oshiwambo, Afrikaans and others. The main spoken languages that had less than 30 children younger than 5 years of age when disaggregated were collapsed into ‘others’. The present study used stratified language as a variable for ethno-linguistic groups.

The main indicator of wealth was adjusted per capita income (APCI). The APCI was classified according to the poverty lines (current Namibian dollar/adult/year). Based on the rates in 2016, the food poor line was N$3517·66 (USD 263·10), the lower bound poverty line (severely poor) was N$4672·00 (USD 349·44), the upper bound poverty line (poor) was N$6249·40 (USD 419·69) and non-poor was above N$6249·40 (USD 419·69).

### Data source

The present study used data extracted from two sources: the NDHS and the most recent NHIES 2015/16. The NDHS collects information on population, health, nutrition and environmental variables^(12)^. It was implemented by the Ministry of Health and Social Services (MoHSS) in collaboration with the Namibia Statistics Agency (NSA) and the National Institute of Pathology. The objective is to provide information for policymaking, planning, monitoring and evaluation at national and regional levels. The NDHS provides anthropometric data on height and weight for the nutritional status of children younger than 5 years. The present study analysed the nutritional status data of children from 1992 to 2013. The 1992, 2000, 2006/7 and 2013 surveys were used to plot child stunting and wasting trends across time in Namibia.

The present study also used the NHIES 2015/16 for a secondary analysis of stunting and wasting prevalence among children younger than 5 years of age. The NHIES is a household survey designed to measure living conditions that is conducted approximately every 5 years by the NSA. The objectives of the NHIES 2015/16 were to measure patterns of consumption and income and other socio-economic indicators. This survey sampled 10 368 households from April 2015 to March 2016. The survey provided representative child anthropometric data at the national level for each of the 14 regions in the country. This was used to assess the prevalence of stunting and wasting in children younger than 5 years of age based on background characteristics, and to compare regional differences in malnutrition.

The two main differences between the data sources lie in the data collection period and the measurement protocol. The NDHS data was collected over a shorter period from May to September, whereas the NHIES collected data across the whole year from April to March of the following year. Also, the measurement protocol of the sources varied between the two sources. The methodology of NDHS was transparent since it specified the procedures for measuring the height/length of the children. Standing height was measured for children older than 2 years and lying length was obtained in children less than 2 years old. However, NHIES did not state details about the measurement protocol in the final report.

The inclusion criteria for our analysis of NHIES anthropometry were children younger than 60 months and measurements of height and weight within the biological parameters – children with out-of-range measurements or missing age were excluded. The analysis for this survey followed the methodology and definitions used in the DHS. Detailed information on NDHS and NHIES sources and methodology is available in the survey reports^(12,15)^.

### Statistics

For our study using the NHIES data, the prevalence of malnutrition in children younger than 5 years of age in Namibia was analysed using two methods: the standard World Health Organization (WHO) flags to remove biologically implausible measurements and the PROBIT method to account for high variance caused by random error. Both methods relied on the use of the 2006 WHO Child Growth Standards. The WHO flags define biological implausibility as a *z*-score lying outside <−6|>6 for HAZ and <−5|>5 for WHZ. PROBIT is an alternative method for estimating prevalence recommended by the WHO when data quality is poor. The PROBIT method assumes that the mean is robust and the sd is not and uses an sd of 1 as a hypothetical value if the data quality is perfect. The PROBIT is (−2 – [observed mean *z*-score])^(16)^.

Analysis of WHO flags was performed using IBM SPSS Statistics for Windows, Version 24.0 (IBM Corp., Armonk, NY, USA). The SPSS macro from the WHO was used to calculate the *z*-scores for HAZ and WHZ. PROBIT analysis relied on a Microsoft Excel spreadsheet (Microsoft, Redwood, Washington, USA) and the mean *z*-scores from the WHO flags. PROBIT is defined by the following function in Microsoft Excel:  = (NORM.DIST(-2,x,1,TRUE)) × 100.

All data on NDHS were extracted from the DHS Program StatComplier^(17)^. Regional prevalence maps according to the threshold were created using ArcGIS (ArcGIS 10.7, Esri, Berkeley, CA, USA).

The background characteristics of HAZ and WHZ were generated using descriptive analysis. The study followed the DHS background characteristics and methodology to disaggregate the estimates of nutritional status. The WHO/UNICEF standards were used for the prevalence thresholds for HAZ and WHZ^(18)^.

### Ethics

This is a secondary analysis of anonymous data where no individual, cluster or village location could be identified; so, formal ethical clearance was not required. The dataset obtained from the NSA is publicly available.

## Results

### Response

**Fig. 1.**
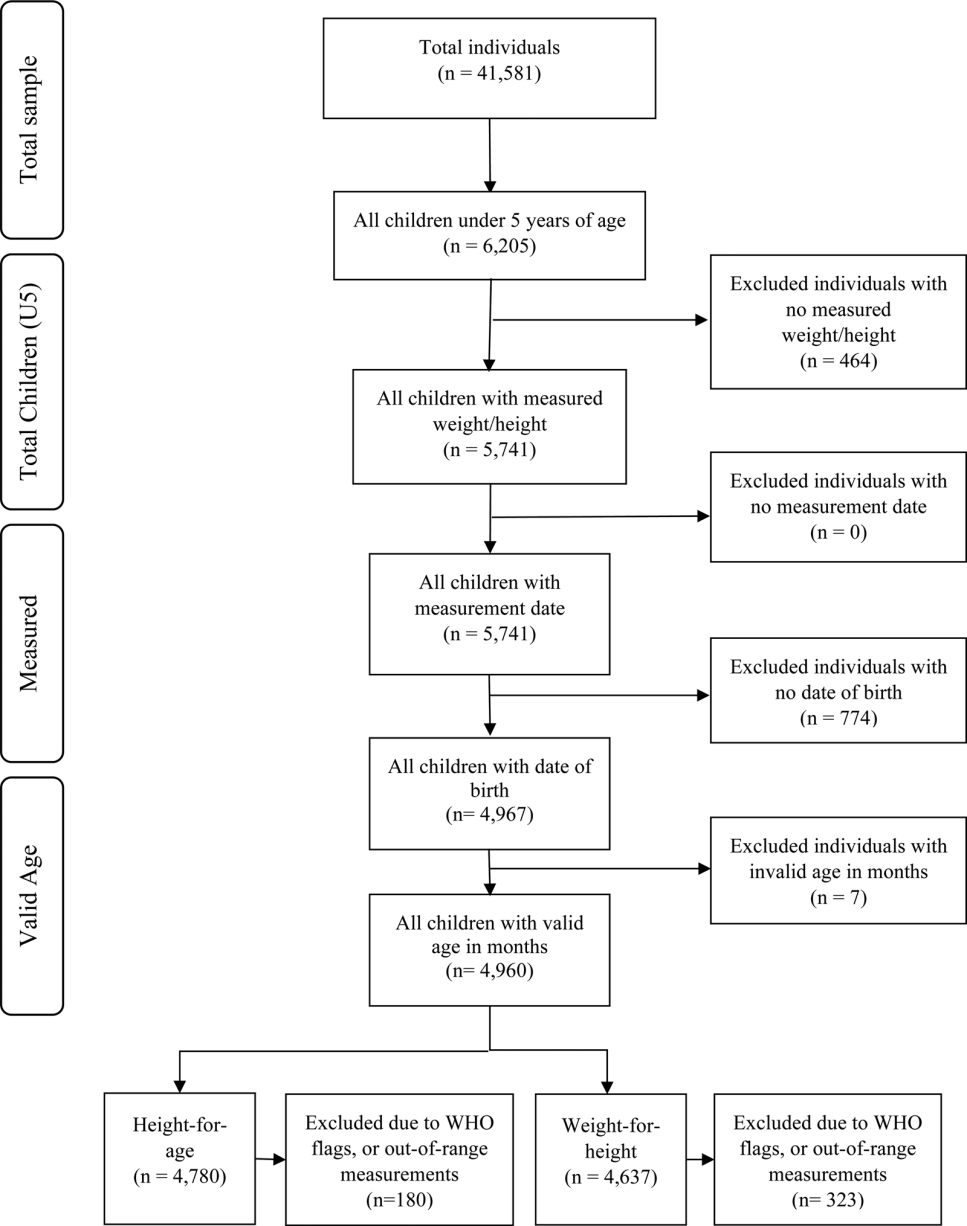
Flowchart of 2015/16 NHIES sample selection.

Fig. 1 shows the flowchart of the NHIES 2015/16 individual dataset, with a total of 41 581 people including 6205 children younger than 5 years of age, who were eligible to be measured. The enumerators measured 5741 children, giving an overall response rate of 92·5 % for child anthropometry. After excluding children with missing measurement dates (*n* 0), date of birth (*n* 774), and with an invalid age in months (n=7), the total number of children remaining for analysis was 4960. The total number of children who were considered for HAZ and WHZ was 4780 and 4637, respectively. The rest were excluded due to WHO flags or out-of-range measurements. The percentage of biologically implausible measurements was 9 %, indicating NHIES collected poor quality child anthropometry.

### Comparing two methods with overall prevalence

Table 1 shows that WHO flags resulted in a higher total stunting prevalence (30·3 %) than PROBIT (20·9 %). In the disaggregated analysis among ethno-linguistic groups, the stunting prevalence was above 60 % with both methods for Khoisan group, whereas the other language groups showed a 32 % or less incidence of stunting. In addition, Khoisan group also displayed the highest wasting prevalence compared to the one of other ethno-linguistic groups, with both the WHO flags (19 %) and the PROBIT method (12·5 %). Furthermore, while WHO flags produced a similarly high prevalence of SAM across ethno-linguistic groups, the PROBIT method indicated that the SAM prevalence in the Khoisan group was three times greater than that in any other group (1·6 % for Khoisan *v*. <0·5 % for all other groups).

**Table 1.** Prevalence of stunting and wasting in children under 5 years with WHO flags and PROBIT method

		Height-for-age^a^					Weight-for-height			
		WHO flags		PROBIT			WHO flags		PROBIT	
Background characteristics	*n*	Below −3 sd (%)	Below −2 SD^b^ (%)	Below −3 sd (%)	Below −2 SD^b^ (%)	*n*	Below −3 sd (%)	Below −2 SD^b^ (%)	Below −3 sd (%)	Below −2 SD^b^ (%)
Age in months										
<6	491	10·1	21·8	0·4	4·6	459	7·2	16·5	0·2	3·3
6–8	252	8·0	15·3	0·2	3·1	249	5·6	14·2	0·4	5·2
9–11	254	10·7	25·3	1·2	10·4	254	4·4	13·0	0·2	3·1
12–17	526	18·3	33·6	4·4	23·9	504	6·3	13·7	0·3	4·2
18–23	465	16·8	38·1	6·3	29·8	460	4·1	9·9	0·1	2·4
24–35	896	16·4	37·3	7·9	34·1	887	3·5	8·9	0·2	3·3
36–47	906	10·9	31·3	5·7	28·1	872	2·9	9·6	0·4	4·6
48–59	990	8·6	27·2	4·0	22·7	952	2·4	10·0	0·6	6·7
Sex										
Male	2464	14·0	32·2	4·3	23·6	2381	3·7	10·7	0·3	3·8
Female	2316	11·0	28·4	2·8	18·1	2256	4·3	11·6	0·4	4·6
Residence										
Urban	2161	10·0	25·4	1·9	14·2	2041	4·7	11·8	0·2	3·3
Rural	2619	14·8	34·4	5·5	27·4	2596	3·5	10·6	0·4	4·9
Region										
Erongo	284	5·6	18·5	1·3	10·9	267	3·8	7·5	0·1	1·5
Hardap	185	17·2	30·1	3·7	21·5	167	5·4	13·7	0·3	4·3
Karas	169	10·3	25·4	1·6	12·7	160	7·1	21·4	0·4	5·1
Kavango East	353	13·7	31·6	3·1	19·5	342	8·7	14·6	0·5	5·7
Kavango West	252	8·6	28·2	2·3	15·9	248	3·9	10·4	0·6	6·2
Khomas	738	10·9	26·7	2·2	15·4	689	5·2	14·1	0·3	3·7
Kunene	206	8·6	27·9	3·1	19·2	202	5·4	8·6	0·3	4·1
Ohangwena	623	17·0	37·6	6·8	31·2	622	2·5	7·7	0·4	4·6
Omaheke	163	8·7	30·4	3·1	19·5	158	1·8	8·4	0·3	3·6
Omusati	532	19·8	42·0	10·2	39·4	533	1·4	9·2	0·3	4·0
Oshana	380	9·9	24·5	3·0	18·9	369	1·7	6·2	0·3	4·2
Oshikoto	386	12·3	29·7	4·1	23·0	380	4·4	15·0	0·7	6·9
Otjozondjupa	315	13·1	31·5	3·1	19·5	312	4·5	13·0	0·3	3·8
Zambezi	193	8·3	24·6	1·7	12·9	187	3·9	10·1	0·1	2·4
Main languages spoken										
Khoisan	79	40·7	68·2	34·8	72·9	81	5·2	19·0	1·6	12·5
Zambezi languages	192	5·9	21·6	1·1	9·9	188	5·3	13·0	0·1	2·4
Otjiherero	446	8·5	27·4	1·9	14·0	421	4·9	11·1	0·2	3·0
Rukavango	702	11·9	28·3	2·7	17·9	681	6·5	13·4	0·5	5·6
Nama/Damara	553	10·4	30·9	3·9	22·4	525	4·9	10·5	0·4	4·6
Oshiwambo	2369	13·9	32·2	4·6	24·5	2323	3·0	10·6	0·4	4·6
Afrikaans	271	12·2	25·8	2·1	15·2	251	4·9	10·3	0·1	1·5
Other	167	9·9	18·6	1·1	9·9	166	0·9	7·6	0·1	1·3
Wealth										
Food poor	289	16·8	37·0	6·3	29·8	277	5·6	17·3	0·8	7·9
Severely poor^c^	566	18·2	41·2	7·4	32·6	545	5·2	15·0	0·6	6·7
Poor^d^	926	18·1	39·0	6·9	31·6	906	4·3	12·7	0·5	5·8
Non-poor	3854	11·3	28·3	2·9	18·7	3731	4·0	10·8	0·3	3·8
Total	4780	12·6	30·3	3·5	20·9	4637	4·0	11·2	0·3	4·2

Table is based on children who stay in the household on the night before the interview. Table is based on children with valid dates on birth (month and year) and valid measurement of both height and weight. Each of the indices is expressed in standard deviation units (sd) from the median of the WHO child growth standards. Wealth is classified with the poverty lines (current Namibian Dollars/adult/year). The food poor line was N$3517·66 (USD 263·10), the lower bound poverty line (severely poor) was N$4672·00 (USD 349·44), the upper bound poverty line (poor) was N$6249·40 (USD 419·69) and non-poor was above N$6249·40 (USD 419·69), based on the rates in 2016.

a Recumbent length is measured for children under age 2 and in the few cases when the age of the child is unknown and the child is less than 85 cm; standing height is measured for all other children.

b Includes children who are below −3SD.

c Includes children who are food poor.

d Includes children who are in severely poor and food poor.

### Comparing two methods with trends over time

**Fig. 2.**
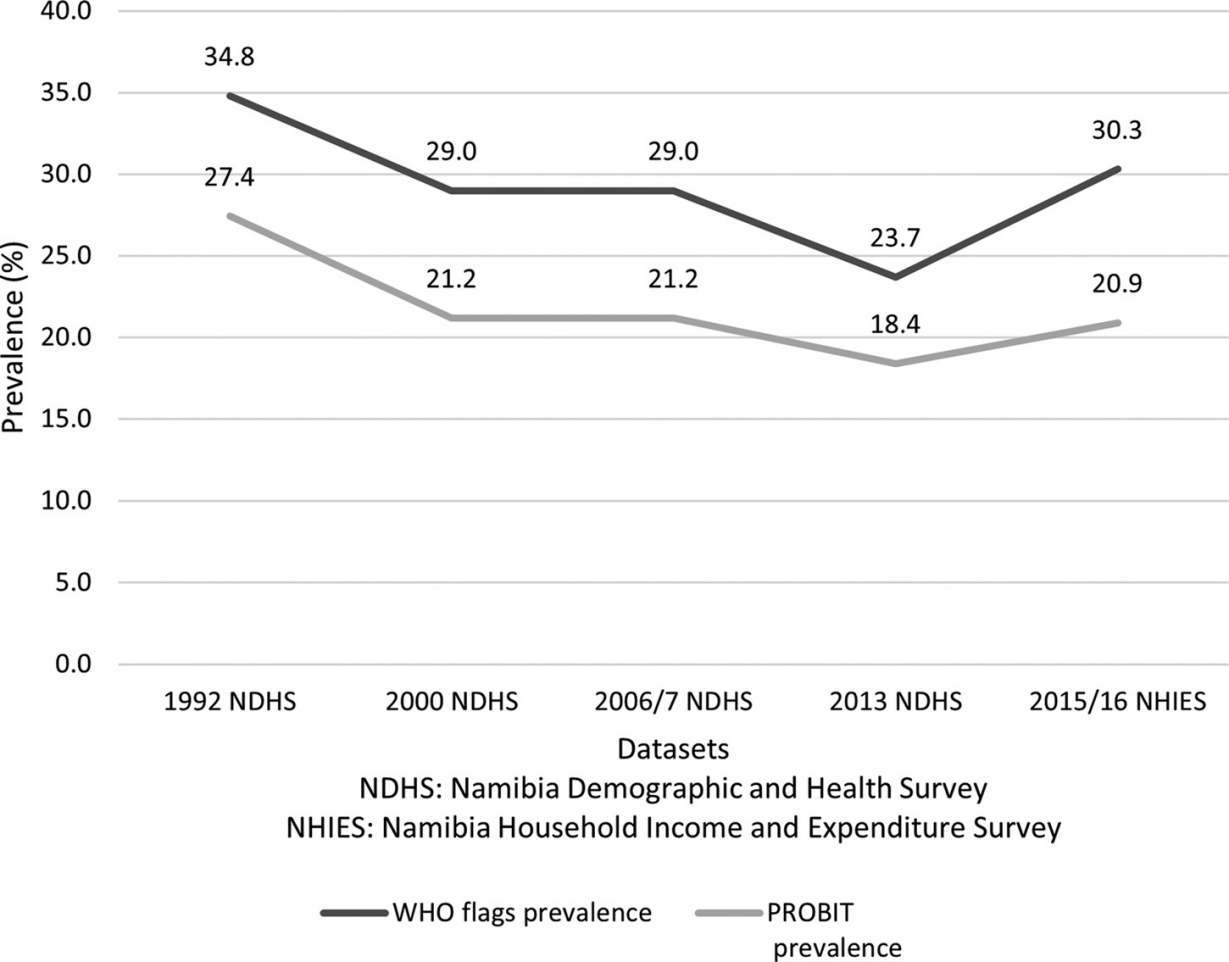
Trends in the national prevalence of stunting among children under age 5 comparing WHO flags and PROBIT method.

**Fig. 3.**
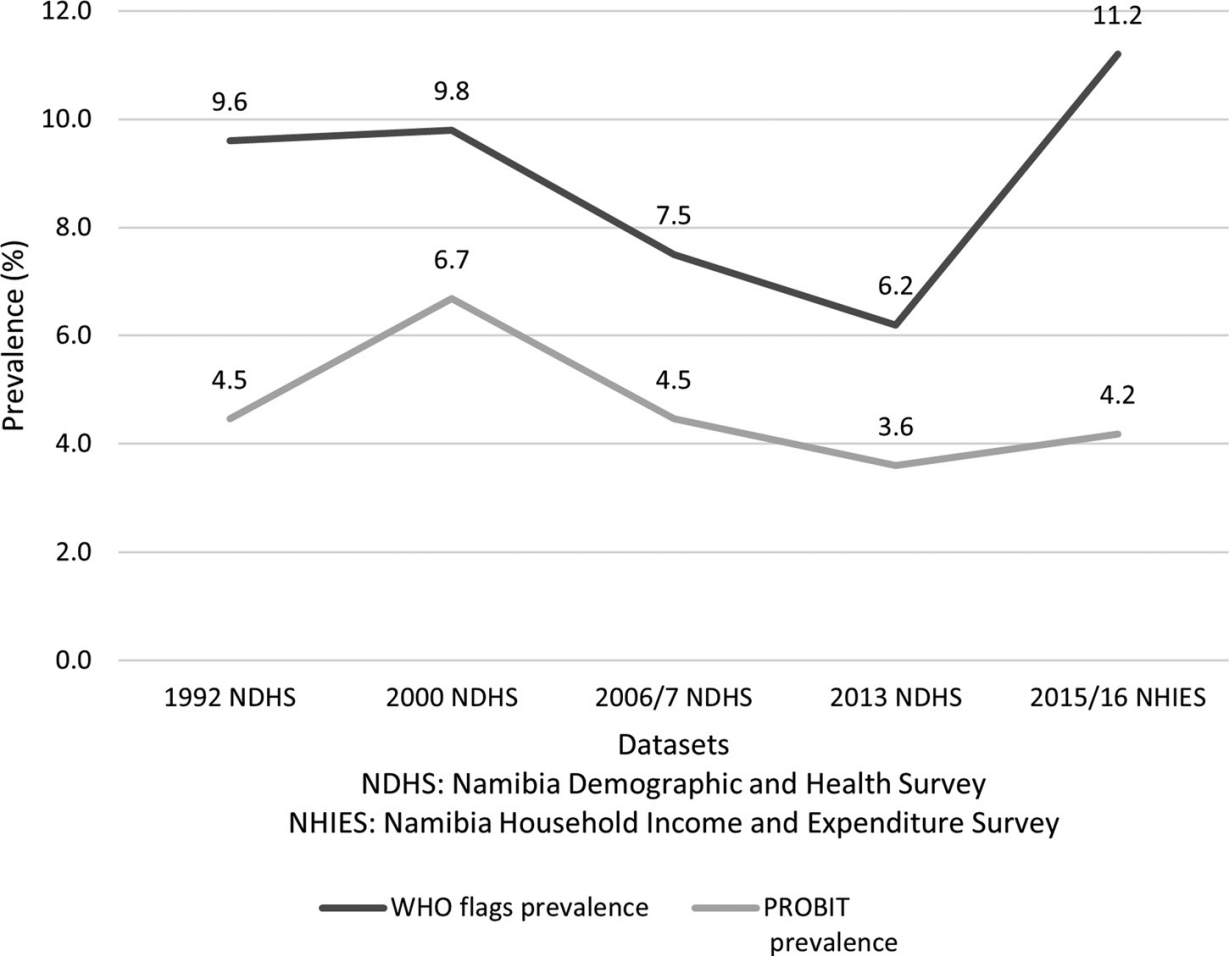
Trends in the national prevalence of wasting among children under age 5 comparing WHO flags and PROBIT method.

We compared the trends in the national prevalence of stunting and wasting among children younger than 5 years of age using WHO flags and the PROBIT method (Figs 2 and 3). These trends present the prevalence of child stunting and wasting across two types of surveys: the NDHS (1992, 2000, 2006/7, 2013) and the NHIES (2015/16). As shown in Fig. 2, both methods demonstrated a similar trend from 1992 to 2013, but the prevalence of stunting was lower across all years with the PROBIT method than with WHO flags. However, the increase in stunting prevalence from 2013 to 2016 was greater with WHO flags (6·6 percentage points) than with the PROBIT method (2·5 percentage points). Fig. 3 shows pronounced differences between the two methods for wasting trends from 2013 to 2016. From 2013 to 2016, the prevalence of child wasting increased by 5·0 percentage points using the WHO flags; in contrast, only 0·6 percentage point increase was seen using the PROBIT method.

### Comparing two methods with targeting

A comparison between WHO flags and PROBIT methods in reporting stunting and wasting prevalence in children younger than 5 years of age in Namibia is shown in Fig. 4. Stunting prevalence thresholds were the following: very low (<2·5 %), low (2·5–<10 %), medium (10–20 %), high (20–<30 %) and very high (≥30 %). Using the WHO flags, six out of the fourteen regions were classified as ‘very high’ for child stunting prevalence (Fig. 4(a)), whereas the PROBIT method identified only two northern regions (Omusati and Ohangwena) (Fig. 4(b)). No regions achieved the ‘very low’ or ‘low’ stunting levels with either method. The figures are available in the Supplementary Material.

Wasting prevalence thresholds were the following: very low (<2·5 %), low (2·5–<5 %), medium (5–10 %), high (10–<15 %) and very high (≥15 %). More than half of the regions were classified as ‘medium’ or ‘higher’ in the prevalence of wasting using WHO flags (eight out of the fourteen regions) (Fig. 5(a)). The PROBIT method, on the other hand, showed that all regions exhibit ‘medium’ to ‘very low’ levels of wasted children (Fig. 5(b)). Four out of the fourteen regions were classified at a ‘medium’ level and the rest achieved ‘very low’ or ‘low’ levels. One region, Karas, shifted from ‘very high’ to ‘medium’ when comparing the results between the WHO flags and the PROBIT method.

## Discussion

### Overview

The present study showed that different analysis methods result in varying stunting and wasting prevalence in children younger than 5 years of age when using the data from national surveys with poor quality child anthropometry. The largest difference between the two methods is in the calculated estimates. The WHO flags resulted in a higher prevalence than the PROBIT method.

The results of the WHO flags were possibly due to the overestimation of measurement errors. When random errors occur in measurements, the sd increases, causing more children to fall into the tails of the distribution; therefore, our results most likely reflected an overestimated prevalence. Previous studies that investigated the cleaning criteria for malnutrition prevalence revealed that the WHO 2006 criteria were the most inclusive, resulting in the highest prevalence among different cleaning criteria^(19)^. Another study argued that even modest random errors can cause a doubling of malnutrition prevalence^(20)^.

PROBIT estimates were smaller because they assume a normal distribution. Since this method estimates the prevalence of malnutrition indirectly by computing the area under the normal distribution curve, it excludes extreme values and produces a smaller standard error for the prevalence estimate than the applications of WHO flags. As a result, the PROBIT estimates were affected to a lesser extent by measurement errors than the WHO flags. These findings are similar to those of a previous study, which analysed the performance of PROBIT and WHO flags and reported that the 95 % confidence interval was lower for the PROBIT method than for WHO flag estimates^(21)^. Our results with the PROBIT method possibly led to less misinterpreted results.

### Implications of using the different methods for the national prevalence and trends

The present study showed that estimation methods affected the reported national prevalence of stunting and wasting from 1992 to 2016. The prevalence of wasting estimated with WHO flags showed an abrupt change indicative of an acute emergency from 2013 to 2016; however, there was only a small increase in acute malnutrition over the same period when estimated by the PROBIT method. According to WHO thresholds, a wasting prevalence of 15 % or more, or a prevalence between 10 and 14 % with exacerbating conditions, requires attention^(18)^. This revealed a problematic trend because the estimate with WHO flags suggested that immediate intervention and supplementary feeding are called for, while the PROBIT method suggested that there is little to no public health emergency. This profound difference in wasting estimates reveals the magnitude of influence the data adjustment has on prevalence and trends. Furthermore, it can potentially lead to inefficient action in the implementation of malnutrition treatment and interventions, depending on which analysis method is utilised.

### Implications of targeting regions with different methods

High-risk regions were easily identified by comparing the regions according to the WHO prevalence thresholds. PROBIT allowed us to compare regions based on the mean *z*-score, which revealed prominent changes in prevalence as well as thresholds in priority regions. A previous study stated that the PROBIT method provided results that were more precise than the WHO estimates, and therefore recommended the former for surveillance and monitoring^(22)^.

The Government of Namibia reported the most detrimental nutritional challenges lie in Northern areas (Oshana, Ohangwena, Omusati, Oshikoto, Kavango and Zambezi)^(23)^. The target regions suggested by the PROBIT method support similar northern regions, while the WHO flags showed high or medium stunting and wasting across the entire country. Since poor quality anthropometry results in overestimation of prevalence, relying solely on WHO flags may result in overestimation of the number of priority areas. Using the PROBIT method when poor quality anthropometry is suspected may help identify regional differences for prioritisation that would have otherwise been masked by high prevalence in all regions when using WHO flags.

### Implications for identifying vulnerable groups

One of the most notable results of the present study was the malnutrition status of ethno-linguistic groups. The Khoisan language group, spoken by Namibia's San ethnic group, exhibited the highest prevalence of stunting with both WHO flags (68·2 %) and PROBIT (72·9 %) methods as compared to other language groups. The San community is the most vulnerable and impoverished group in the country^(24)^. Even when compared to Burundi, which has the world's highest prevalence of stunting (57·7 %), the San had surpassed these estimates^(25)^. For SAM estimates, ethno-linguistic groups resulted in little difference across the groups with WHO flags. However, with the PROBIT method, the San would be a priority target group for therapeutic feeding because SAM prevalence was three times higher than any other group.

As Dale *et al.* reported, the PROBIT method is argued to be more advantageous for smaller sample sizes of less than 150 compared to a larger sample size^(22)^. A study by Blanton *et al.* also reported the advantages of the PROBIT method for lower sample sizes^(21)^. Of the ethno-linguistic groups, San was the only group with a sample size of less than 150, and this was the only occasion where the PROBIT method displayed a greater estimate than the WHO flag prevalence. In addition, a higher SAM prevalence among the San was completely masked when using WHO flags. Therefore, the San estimates could have better reflected the characteristics of the PROBIT method because of the smaller sample size than that of the other ethno-linguistic groups.

### Implications for caseload and coverage estimation

The NHIES findings resulted in different caseloads for treating SAM, with the WHO estimate (4·0 %) being 13 times greater than the PROBIT estimate (0·3 %). Previous literature suggests that caseload estimates from SAM prevalence should be used with caution for services and policies^(26)^. Similar results have been found, where the precision of estimates of caseload and burden was improved by using PROBIT to estimate the prevalence of SAM^(27)^. Therefore, the present study shows that the use of PROBIT to avoid overestimation of caseload is particularly important when using surveys with poor quality child anthropometry.

### Way forward

The true prevalence is more likely to be accurately represented through high-quality surveys with consistent and intensive field supervision during data collection. Considering the differing data qualities among DHS across countries, extensive training and adequate supervision can improve the overall standard and achieve higher quality anthropometric data^(28,29)^. During field work, quality assurance procedures are recommended such as measuring each child twice and taking the average, and a practical test on measurement as part of an extensive training of data collectors and incorporating in-process quality checks for intra- and inter-observer technical error of measurement^(30,31)^.

The present study highlights the importance of high-quality anthropometric data in public health and is relevant to other low- and middle-income countries with similar data quality issues in child anthropometry. However, what constitutes poor data quality and ways to standardise poor data adjustments are still an ongoing challenge and a topic of debate. There are limitations in using the PROBIT method because it assumes that the population has a normal distribution. Given Namibia's extreme inequality, a normal distribution with an sd of 1 may not properly represent the true population of the country. Additionally, there is no consensus on the sample size for which the PROBIT method works well.

After comparing the two methods of analysis for calculation of malnutrition prevalence, it was revealed that the prevalence estimates, trends, target regions and vulnerable groups varied with the analysis method. The findings presented the importance of data adjustment for surveys with poor data quality, and how poor quality can mask important differences and lead to incorrect interpretations. In surveys with minimal measurement error, the use of the standard method of removing biologically implausible measurements with WHO flags is sufficient to capture and remove outliers caused by human error. However, the use of WHO flags does not adequately remove the overdispersion caused by frequent, random measurement error; which means that prevalence is typically overestimated in surveys with poor quality anthropometry. The present study supports the recommendation to use PROBIT to analyse poor quality anthropometry data. Despite the existing WHO recommendation, the PROBIT method is not often used to re-analyse poor quality child anthropometry. The present study showed that the PROBIT method can make poor quality data useful, helping to identify high-risk populations, meaningfully assess trends and effectively plan service delivery.

## Supplementary material

For supplementary material accompanying this paper visit https://doi.org/10.1017/jns.2022.67. click here to view supplementary material

## References

[1] Assaf S, Kothari MT & Pullum TW (2015) An Assessment of the Quality of DHS Anthropometric Data, 2005–2014. Rockville, MD, USA: ICF International.

[2] Corsi DJ, Perkins JM & Subramanian S (2017) Child anthropometry data quality from Demographic and Health Surveys, Multiple Indicator Cluster Surveys, and National Nutrition Surveys in the West Central Africa region: are we comparing apples and oranges? Global Health Action 10, 1328185. 28641057 10.1080/16549716.2017.1328185 PMC5496063

[3] Shireen A, Kothari M & Pullum T (2015) An Assessment of the Quality of DHS Anthropometric Data, 2005–2014. DHS Methodological Reports No 16. Rockville, MD, USA: ICF International.

[4] World Health Organization (2019) Recommendations for Data Collection, Analysis and Reporting on Anthropometric Indicators in Children Under 5 Years Old. Geneva: World Health Organization.

[5] Mei Z & Grummer-Strawn LM (2007) Standard deviation of anthropometric Z-scores as a data quality assessment tool using the 2006 WHO growth standards: a cross country analysis. Bull World Health Organ 85, 441–448. 17639241 10.2471/BLT.06.034421 PMC2636355

[6] World Health Organization (1997) WHO Global Database on Child Growth and Malnutrition. Geneva: World Health Organization.

[7] Perumal N, Namaste S, Qamar H, (2020) Anthropometric data quality assessment in multisurvey studies of child growth. Am J Clin Nutr 112, 806S–815S. 32672330 10.1093/ajcn/nqaa162 PMC7487428

[8] Lyons-Amos M & Stones T (2017) Trends in Demographic and Health Survey data quality: an analysis of age heaping over time in 34 countries in Sub-Saharan Africa between 1987 and 2015. BMC Res Notes 10, 1–7. 29262857 10.1186/s13104-017-3091-x PMC5738749

[9] Betts P, Voss L & Bailey B (1992) Measuring the heights of very young children. BMJ 304, 1351. 1611333 10.1136/bmj.304.6838.1351 PMC1882034

[10] WHO Multicentre Growth Reference Study Group & de Onis M (2006) Reliability of anthropometric measurements in the WHO Multicentre Growth Reference Study. Acta Paediatr Suppl 95, 38–46. 16817677 10.1111/j.1651-2227.2006.tb02374.x

[11] The World Bank (2022) *Gini Index (World Bank Estimate)*. https://data.worldbank.org/indicator/SI.POV.GINI (accessed 31 March 2022).

[12] The Nambia Ministry of Health and Social Services (MoHSS) & ICF International (2014) The Namibia Demographic and Health Survey 2013. Windhoek, Namibia, and Rockville, MD, USA: The Nambia Ministry of Health and Social Services (MoHSS), ICF International.

[13] Stell G & Fox T (2015) Ethnicity in discourse: the interactional negotiation of ethnic boundaries in post-apartheid Namibia. Ethn Racial Stud 38, 976–992.

[14] Mchombu KJ & Mchombu CM (2014) The role of information and knowledge in poverty eradication in Africa: a case study of Namibia. In IFLA WLIC 2014 - Lyon - Libraries, Citizens, Societies: Confluence for Knowledge in Session 189 - Access to Information Network - Africa (ATINA) Special Interest Group. 16-22 August 2014. France: Lyon.

[15] Namibia Statistics Agency (2018) Namibia Household Income and Expenditure Survey (NHIES) 2015/16 Report. Windhoek: Namibia Statistics Agency.

[16] WHO (1995) Physical Status: The Use and Interpretation of Anthropometry. Report of a WHO Expert Committee. WHO Technical Report Series 854. Geneva: World Health Organization. 8594834

[17] ICF International (2012) The DHS Program STATcompiler. http://www.statcompiler.com (accessed 31 March 2022).

[18] De Onis M, Borghi E, Arimond M, (2019) Prevalence thresholds for wasting, overweight and stunting in children under 5 years. Public Health Nutr 22, 175–179. 30296964 10.1017/S1368980018002434 PMC6390397

[19] Crowe S, Seal A, Grijalva-Eternod C, (2014) Effect of nutrition survey ‘cleaning criteria’ on estimates of malnutrition prevalence and disease burden: secondary data analysis. PeerJ 2, e380. 24883244 10.7717/peerj.380 PMC4034601

[20] Grellety E & Golden MH (2016) The effect of random error on diagnostic accuracy illustrated with the anthropometric diagnosis of malnutrition. PLoS ONE 11, e0168585. 28030627 10.1371/journal.pone.0168585 PMC5193513

[21] Blanton CJ & Bilukha OO (2013) The PROBIT approach in estimating the prevalence of wasting: revisiting bias and precision. Emerg Themes Epidemiol 10, 8. 23981669 10.1186/1742-7622-10-8 PMC3846578

[22] Dale NM, Myatt M, Prudhon C, (2013) Assessment of the PROBIT approach for estimating the prevalence of global, moderate and severe acute malnutrition from population surveys. Public Health Nutr 16, 858–863. 23174145 10.1017/S1368980012003345 PMC10271864

[23] World Health Organization (2012) Landscape Analysis to Accelerate Actions to Improve Maternal and Child Nutrition in Namibia. Windhoek, Namibia: Republic of Namibia.

[24] Suzman J (2001) An Assessment of the Status of the San in Namibia. *Regional Assessment of the Status of the San in Southern Africa*, vol. 4. Windhoek, Namibia: Legal Assistance Centre.

[25] Akombi BJ, Agho KE, Merom D, (2017) Child malnutrition in Sub-Saharan Africa: a meta-analysis of demographic and health surveys (2006–2016). PLoS ONE 12, e0177338. 28494007 10.1371/journal.pone.0177338 PMC5426674

[26] Deconinck H, Pesonen A, Hallarou M, (2016) Challenges of estimating the annual caseload of severe acute malnutrition: the case of Niger. PLoS ONE 11, e0162534. 27606677 10.1371/journal.pone.0162534 PMC5015826

[27] Bulti A, Briend A, Dale NM, (2017) Improving estimates of the burden of severe acute malnutrition and predictions of caseload for programs treating severe acute malnutrition: experiences from Nigeria. Arch Public Health 75, 66. 29152260 10.1186/s13690-017-0234-4 PMC5679511

[28] Bilukha O, Couture A, McCain K, (2020) Comparison of anthropometric data quality in children aged 6–23 and 24–59 months: lessons from population-representative surveys from humanitarian settings. BMC Nutr 6, 1–12. 33292633 10.1186/s40795-020-00385-0 PMC7664017

[29] Leidman E, Mwirigi LM, Maina-Gathigi L, (2018) Assessment of anthropometric data following investments to ensure quality: Kenya Demographic Health Surveys case study, 2008 to 2009 and 2014. Food Nutr Bull 39, 406–419. 30037280 10.1177/0379572118783181 PMC6327319

[30] Stomfai S, Ahrens W, Bammann K, (2011) Intra- and inter-observer reliability in anthropometric measurements in children. Int J Obes (Lond) 35, S45–S51. 10.1038/ijo.2011.34 21483422

[31] Jamaiyah H, Geeta A, Safiza M, (2010) Reliability, technical error of measurements and validity of length and weight measurements for children under two years old in Malaysia. Med J Malaysia 65, 131–137. 21488474

